# Descriptive assessment of rabies post-exposure prophylaxis procurement, distribution, monitoring, and reporting in four Asian countries: Bangladesh, Bhutan, Cambodia, and Sri Lanka, 2017–2018

**DOI:** 10.1016/j.vaccine.2018.10.011

**Published:** 2018-10-09

**Authors:** Anyie J. Li, Nandini Sreenivasan, Umme Ruman Siddiqi, Sanya Tahmina, Kinley Penjor, Ly Sovann, Amila Gunesekera, Jesse D. Blanton, Lea Knopf, Terri B. Hyde

**Affiliations:** a PHI/CDC Global Health Fellowship and ASPPH/CDC Allan Rosenfield Global Health Fellowship, Atlanta, USA; b Centers for Disease Control and Prevention, Atlanta, USA; c Disease Control Unit, Communicable Disease Control, Directorate General of Health Services, Dhaka, Bangladesh; d Dewathang Military Hospital, Department of Medical Services, Ministry of Health, Thimphu, Bhutan; e Communicable Disease Control Department, Ministry of Health, Phnom Penh, Cambodia; f Ministry of Health, Colombo, Sri Lanka; g World Health Organization, Geneva, Switzerland

**Keywords:** Rabies virus, Human rabies, Rabies post-exposure prophylaxis, Rabies vaccine

## Abstract

**Background:**

There are approximately 35,000 human deaths from rabies in Asia annually. Rabies can be prevented through timely post-exposure prophylaxis (PEP) consisting of wound washing, rabies vaccine, and in some cases, rabies immunoglobulin (RIG). However, access to rabies PEP often remains limited to urban areas and is cost-prohibitive. There is little information on procurement, distribution, monitoring, and reporting of rabies PEP.

**Methods:**

We interviewed key informants in the public sector from various levels in Bangladesh, Bhutan, Cambodia, and Sri Lanka between March 2017 and May 2018 using a descriptive assessment tool to obtain information on procurement, distribution, monitoring, and reporting of rabies PEP. These four countries in Asia were chosen to showcase a range of rabies PEP systems. National rabies focal points were interviewed in each country and focal points helped identify additional key informants at lower levels.

**Results:**

A total of 22 key informants were interviewed at various levels (central level to health facility level) including national rabies focal points in each country. Each country has a unique system for managing rabies PEP procurement, distribution, monitoring, and reporting. There are varying levels of PEP access for those with potential rabies exposures. Rabies PEP is available in select health facilities throughout the country in Bangladesh, Bhutan, and Sri Lanka. In Cambodia, rabies PEP is limited to two urban centers. The availability of RIG in all four countries is limited. In these four countries, most aspects of the rabies PEP distribution system operate independently of systems for other vaccines. However, in Bhutan, rabies PEP and Expanded Programme on Immunization (EPI) vaccines share cold chain space in some locations at the lowest level. All countries have a monitoring system in place, but there is limited reporting of data, particularly to the central level.

**Conclusion:**

Systems to procure, deliver, monitor, and report on rabies PEP are variable across countries. Sharing information on practices more widely among countries can help programs to increase access to this life-saving treatment.

## 1. Introduction

Rabies is caused by a lyssavirus and is nearly 100% fatal once clinical symptoms occur. Worldwide, rabies causes an estimated 59,000 human deaths annually with approximately 60% of cases occurring in Asia [1]. The total number of deaths is likely an underestimate due to poor surveillance and reporting, and a lack of laboratory testing capacity [1–3].

Rabies in humans can be prevented through timely and appropriate post-exposure prophylaxis (PEP). Rabies PEP consists of wound washing, immediate rabies vaccination following the potential exposure, and in some cases, rabies immunoglobulin (RIG). Currently only cell-culture or embryonated egg-based rabies vaccines (CCEEVs) are recommended by the World Health Organization (WHO); the original nerve-tissue based vaccine (NTBV) has not been recommended since the 1980s [3,4]. Very few countries in the world continue to use NTBV.

**Fig. 1.**
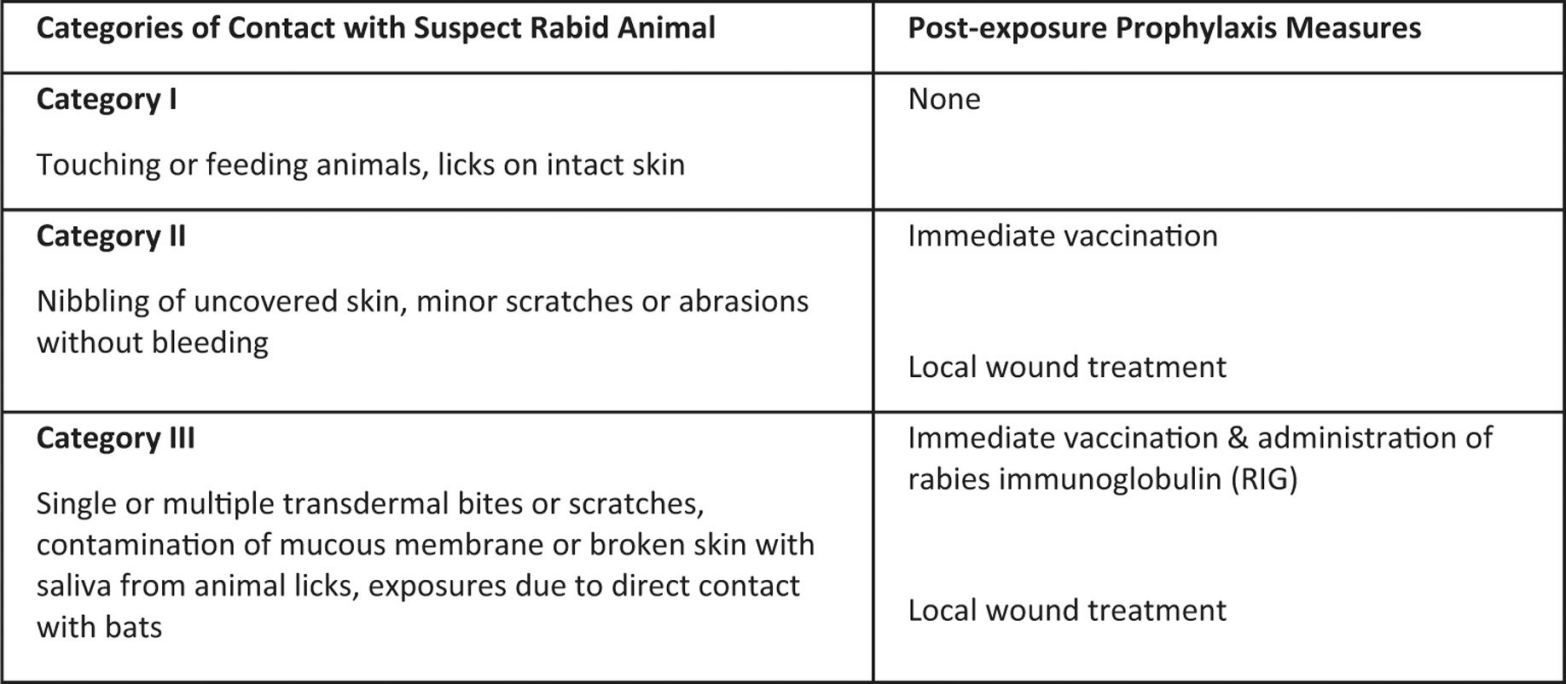
Categories of contact with suspect rabid animal and post-exposure prophylaxis (PEP) [3,8].

WHO provides guidance for rabies PEP according to three categories of bites (Fig. 1) [3]. Rabies vaccine is recommended for all Category II and III bites, whereas RIG is only recommended for Category III bites; however, less than 2% of patients worldwide who need RIG actually receive it [3,5–8]. Rabies vaccine can be administered by the intramuscular (IM) route, using a full vaccine vial per patient, or by the intradermal (ID) route, using a fractional dose [8]. Vaccination schedules and dosage vary by route of administration [8]. Fractional ID administration is cost-, dose-, and time-sparing as compared to IM administration [8]. Without PEP, an estimated 3 million people worldwide would die from rabies annually [1].

In 2015, WHO, the World Organization for Animal Health (OIE), the Food and Agriculture Organization of the United Nations (FAO) and the Global Alliance for Rabies Control (GARC) established a global goal of zero dog-mediated human rabies deaths by 2030[9]. The Association of Southeast Asian Nations (ASEAN) and the Plus Three Countries (China, Japan, and Korea) developed a regional strategy to eliminate rabies by 2020 and the WHO Southeast Asia Region developed a regional strategy to eliminate human rabies transmitted by dogs by 2020 [10,11]. Sri Lanka developed a national strategy to eliminate human deaths by rabies by 2020 [12]. All strategies include collaboration among partners and illustrate the “One Health” approach in which the animal and human health sectors work together to achieve the goal. While rabies PEP is effective in preventing human rabies deaths, animal rabies control is the solution for rabies elimination and animal rabies control is a main pillar of each of these strategies. However, there have been varying degrees of progress toward reaching these goals. For human rabies prevention, access to rabies PEP continues to be cost-prohibitive and availability is largely restricted to urban areas.

Knowledge on rabies PEP procurement and distribution is limited, and there is little information on monitoring and reporting of its use. This project was part of a larger global project conducted in 23 countries to better understand rabies PEP systems; results can be found in *Overview of rabies post-exposure prophylaxis access, procurement and distribution in selected countries in Asia and Africa, 2017–2018* (Sreenivasan, unpublished results). Bangladesh, Bhutan, Cambodia, and Sri Lanka were chosen for in-depth assessments to highlight unique aspects of delivery and distribution systems in Asia. We aimed to include countries with varying burdens of human rabies and varying levels of effort to control and eliminate rabies, as well as countries in both the Southeast Asia and Western Pacific WHO regions and countries in various categories of eligibility for support from Gavi, the Vaccine Alliance (eligible, non-eligible and transitioning) [11–21]. This paper outlines procurement, distribution, monitoring, and reporting systems of rabies PEP in four countries with diverse delivery systems in the Asia region (Bangladesh, Bhutan, Cambodia, and Sri Lanka), with an emphasis on the vaccine component of PEP, and aims to summarize practices from each country in order to identify areas for improving access to PEP.

## 2. Methods

We interviewed key informants to obtain information on procurement, distribution, monitoring, and reporting of rabies PEP, using a descriptive assessment tool. The tool consisted of ten categories of questions on program delivery, vaccine procurement, demand, distribution, cold-chain and vaccine storage, forecasting, monitoring, utilization, and reporting. Key informants were identified at each level of the healthcare system based on the structure of the country’s healthcare and rabies PEP system (e.g. central, province, district, health facility) and included national rabies focal points and health facility heads. When possible, we also interviewed other key informants such as Expanded Programme on Immunization (EPI) focal points to gain a more comprehensive understanding of immunization programs in the countries. Additionally, we reviewed vaccination registers to understand the monitoring system of rabies PEP and type of information recorded on bite patients and evaluated cold chain capacity at health facilities visited. We collected data between March 2017 and May 2018. To provide background information on the rabies situation in each country, we also reviewed WHO documents and published studies on rabies relevant to the selected countries.

## 3. Results

A total of 22 individuals were interviewed in the four countries. In each country, the national rabies focal point was interviewed, some with over 20 years of experience in the role. Other individuals interviewed included national-level staff working on rabies (5), WHO country office rabies focal points (2), national-level EPI staff (2), WHO country office EPI staff (2), and health facility heads and health workers (7). In some countries, health facilities were not visited specifically for this assessment and thus are not included in the total number interviewed, but were visited by national rabies focal points as part of their routine work. Table 1 provides an overview of the main aspects of each rabies PEP system from the country assessments, with additional description provided for each country below.

### 3.1. Bangladesh

In Bangladesh, there are an estimated 2000 deaths due to rabies with over 166,000 animal bite victims, the majority of which are dog-bite victims, annually [15]. Rabies became a national priority in Bangladesh in 2011 with the phase-out of NTBV and introduction of CCEEV administered using the ID route, establishment of an elimination strategy through a “One Health” approach, strong political commitment and budget allocation, and regular large-scale animal vaccination program [15,17,18,22].

Rabies PEP is offered at 66 public District Rabies Prevention and Control Centers (DRPCCs) around the country with at least one center in each of the 64 districts and at a National Rabies Prevention and Control Center (NRPCC). The NRPCC provides treatment to approximately 500 patients daily [23,24]. At public treatment centers, patients do not pay for vaccine or RIG, but pay a small amount for a disposable syringe (US$ 0.13). If rabies PEP is out of stock at a public facility, patients must purchase it at a pharmacy (US$ 8.25 per vaccine vial and US$ 12.50 per vial of RIG). The cost of the vaccine can be shared among several patients, as the ID administration route allows one vial to provide several doses [25].

The Ministry of Health (MoH) uses an open bidding system to select the vaccine provided in public facilities. The timeframe from bidding to delivery is six months to one year. Central level procurement is based on the previous years’ consumption through the Communicable Disease Control (CDC) operational plan of the Directorate General of Health Services (DGHS). Since 2014, CDC/DGHS has procured 20–30% more vaccine each year than the previous year, but PEP demand continues to exceed the supply due to increasing public awareness. CDC/DGHS strives to fulfill 100% of the demand year-round at the NRPCC, and 50–80% of the demand at DRPCCs. Rabies focal points from the DRPCCs are responsible for collecting vaccine monthly from the central medical store. Collection requires prior approval for the amount requested from CDC/DGHS and a report on the previous month’s utilization. Focal points are responsible for transporting vaccine back to treatment centers using cold boxes and ensuring storage of vaccines in a refrigerator at the DRPCCs. This system is separate from the EPI program as the EPI program transports routine vaccines from the capital to regional centers and then to the local level using their own mechanisms.

CDC/DGHS provides a standardized register to track patients, record demographics, and monitor vaccine usage, as well as a standardized rabies vaccination card for patients. Patients are also given a reminder card for future appointments. The NRPCC and DRPCCs are required to report the total number of doses and the dates of administration to CDC/DGHS through the National Health Management Information System (MIS-DHIS2) on a monthly basis and reports are aggregated at the central level for planning purposes. The NRPCC and DRPCCs also submit a hard-copy report detailing patient demographic and bite-related information to CDC/DGHS for future planning purposes. There is no formal system for monitoring and reporting adverse events following rabies PEP; instead, information on adverse events is collected and reported ad hoc to CDC/DGHS. There is currently no follow-up of defaulters and individual vaccine completion rates are not checked. However, CDC/DGHS is establishing a regular mobile phone-based monitoring system to identify adverse events and to detect reasons for incompletion of the vaccination schedule.

### 3.2. Bhutan

In Bhutan there were over 7900 reported dog bites and one rabies death in the country in 2016 [26,27]. The Bhutan Ministry of Health updated their National Rabies Management Guidelines in 2014 [28]. Bhutan has emphasized a “One Health” approach bringing together both dog and human rabies prevention and control strategies [14,28,29].

Bhutan’s healthcare system consists of one national, two regional-level, and 30 district hospitals, and 210 basic health units (BHU-I and II), as well as indigenous and outreach clinics throughout the country [27]. Rabies vaccine is provided free of charge at BHU-II or higher health facilities and is administered using the ID route [29]. RIG is provided free of charge on a more limited basis at BHU-I or higher facilities due to high costs. According to Bhutan’s rabies management guidelines, all Category III patients should receive RIG but because of the limited supply due to the high cost, the majority of these patients are not given RIG [8,28,29]. In line with Bhutan’s integrated service delivery policy, rabies vaccine is given by the same staff as EPI vaccines; staff are trained by both programs. Rabies PEP is unavailable in the private sector.

All drugs including rabies vaccines are procured through the Medical Procurement and Supplies Division (MSPD) of the MoH while EPI vaccines are procured by Vaccine Preventable Disease Control Program (VPDP) through UNICEF. Head pharmacy technicians submit annual rabies vaccine requests based on the previous year’s consumption with a one-month buffer. Requests are submitted to the Health Care and Diagnostic Division (HCDD) using a standardized form for all drugs. The HCDD verifies the quantities of rabies vaccine requested and submits the request to the MSPD. The MSPD receives rabies vaccines and they are then distributed in a cold chain van to the hospital level. BHUs then request and collect rabies vaccine from their respective district hospitals as needed. Within some health facilities, rabies vaccines and EPI vaccines are stored together. Rabies vaccine distribution was previously integrated with EPI vaccines as an interim measure until resources were made available to separate the two systems.

All health facilities that provide rabies PEP are required to maintain a vaccine inventory stock ledger and rabies vaccine register with demographics, information on exposure, and vaccination dates. However, there is no mandatory reporting requirement or system to report rabies PEP data, nor are the data monitored at the central level [29]. Vaccination cards included with the vaccine vials are given to patients when available. There is no system for following-up with defaulting patients or documenting individual series completion rates unless the patient is bitten by a confirmed rabid dog. These patients are followed-up by a phone call if they do not return for their additional vaccine doses. Suspected adverse events following PEP are reported using the Adverse Drug Reactions (ADR) form to the Pharmacovigilance unit within the Drug Regulatory Agency; this system is also used for other drugs and vaccines.

### 3.3. Cambodia

There are an estimated 800 human rabies deaths annually in Cambodia with over 700,000 people bitten by dogs each year; dog vaccination campaigns are not conducted regularly [14,30]. There is no national rabies control program but a national strategy is under development.

Three non-private entities provide rabies PEP in Cambodia: Angkor Hospital for Children (AHC) in Siem Reap and the Institut Pasteur du Cambodge (IPC) rabies vaccination center and National Institutes of Public Health (NIPH) clinic in Phnom Penh. Children ≤16 years can receive PEP free of cost at the AHC. Patients of all ages can access rabies vaccine (US$ 12 for the full course) and RIG (US$ 37) at subsidized costs at IPC in Phnom Penh. At both AHC and IPC, RIG is only available in limited quantities and patients are prioritized based on status of dog, wound location, and age. IPC receives approximately 22,000 new patients for PEP annually and AHC receives around 3000 patients. Rabies vaccine is available at full cost (US$ 8–15 per dose depending on brand) at the NIPH clinic; RIG is not available. The NIPH clinic receives around 500 new patients each year. Rabies vaccine is administered using the ID administration route at AHC and IPC and intramuscular (IM) administration route at NIPH clinic. Additionally, rabies vaccine is widely available at a number of private clinics throughout the country, with some also offering RIG. Public health facilities outside of Phnom Penh will often refer adult patients to the private sector for treatment. However, there is limited knowledge of the availability, quality, and cost of PEP at these private clinics.

All three clinics have a procurement focal point. Forecasting is done based on the previous month’s (or several months’) consumption. Vaccines and RIG are purchased directly from a supplier in Phnom Penh and delivered directly to the hospital or clinic, as needed. Vaccines are stored in refrigerators either in the immunization room or in a store room and transferred to a vaccine carrier for daily use.

The NIPH clinic and IPC use electronic databases for stock monitoring and reporting with standardized information collected on patients including demographics, vaccine brand, administration route, WHO bite category, status of dog, and PEP vaccination dates. AHC records similar information in a hard-copy stock ledger and patient chart. Each facility provides their own vaccination card to their patients. There are no mandatory reporting requirements of rabies PEP or patients receiving rabies PEP to the MoH. The NIPH clinic reports the numbers of people receiving rabies vaccine at the clinic to NIPH monthly. NIPH then reports the numbers to MoH. IPC reports annually to the MoH or when requested. AHC does not report to the MOH on rabies vaccination. There is limited defaulter tracking in all facilities with follow-up done ad hoc and only for those with probable rabies exposures (e.g. confirmed rabid biting animal), and no system for monitoring of adverse events.

### 3.4. Sri Lanka

Sri Lanka has had a national rabies control program since 1975 and human rabies has been a notifiable disease since 1971 [12,16,31]. Each year approximately 150,000 people are bit by an animal, the majority of which are dogs, but the government’s commitment to rabies control has resulted in substantial progress in reducing rabies deaths to 20 deaths reported in 2016, as compared to 50–60 deaths reported annually for much of the 2000s [12,31–33]. This progress is attributed to measures such as making rabies a national health priority, providing PEP for free, and animal-sector efforts including dog vaccination [12,13,16].

Rabies PEP is provided for free at the 204 rabies units located in hospitals throughout the country. Higher volume hospitals carry both vaccine and RIG; lower levels only carry rabies vaccine. Rabies units have at least one bed and one computer for electronic reporting, and are staffed with doctors and nurses trained on PEP protocols. PEP is also available in the private sector on a limited basis and at high cost.

Rabies PEP is procured at the central level by the State Pharmaceutical Corporation of Sri Lanka (SPC) and arrives in three stocks annually. SPC is required to procure WHO pre-qualified vaccines. Each rabies unit prepares an annual request based on the previous year’s vaccine use. All rabies units use the same standardized form as for other drug and vaccine requests. Rabies PEP are transported from the central to the regional to the district level either by refrigerator truck or by cold box throughout the year according to a delivery plan decided by the government. Within rabies units, PEP is stored in its own refrigerator. Within emergency rooms, it is stored in ward refrigerators, along with other non-rabies biologicals. Rabies PEP arrives in the country throughout the year, but is often issued to health facilities in smaller stocks because of space shortages. Additional stocks are kept at the district or central level, and sent to facilities as needed.

Each unit maintains an electronic or paper-based patient register with information on patient demographics, animal involved and status, and dates of vaccination. Some rabies units have transitioned to a real-time online data reporting system with other units expected to enhance reporting systems soon. All patients receive a printed PEP card with the dates of subsequent visits. Each unit also has a register for stock monitoring showing daily, weekly, monthly, and yearly balances. Rabies units are required to report on rabies PEP stock and the numbers of individuals receiving PEP monthly to the anti-rabies campaign at the MoH. Defaulter tracking is done for all patients that are considered high risk based on the location or severity of the wound and biting animal status. If a patient is considered high risk, follow-up is done by phone or in-person by a rabies public health inspector (one per district). Adverse events following rabies vaccination is reported through the adverse event reporting system, as with other vaccines.

## 4. Discussion

The countries described have differently structured systems for rabies vaccine procurement, distribution, monitoring, and reporting. Bangladesh, Bhutan, Cambodia, and Sri Lanka also had varying levels of access to rabies PEP for persons with potential exposure. Because of the low numbers of patients requiring rabies PEP, as compared to other types of treatments, PEP can be made accessible without being available at every level of the healthcare system or every health facility [34]. In Bangladesh, Bhutan, and Sri Lanka, rabies vaccine is available at selected health facilities in every district either integrated into existing health services or in designated rabies units. In addition to providing physical access, Bangladesh, Bhutan, and Sri Lanka provide rabies vaccine free or at a nominal cost within the public sector. Accessibility and low cost in these countries may also be partially attributed to the presence of a national rabies program.

Throughout the four countries, availability of RIG is limited, and is cost-prohibitive to governments and patients. When available, its use is often prioritized for young children, patients with severe wounds or wounds at highly innervated body parts, or in exposures to probable or confirmed rabid animals. This prioritization is in line with the 2018 WHO position on rabies immunization emphasizing dose- and cost-sparing approaches [8].

Rabies vaccine distribution systems in these four countries primarily operate independently of systems for other vaccines. Bangladesh’s distribution system requires district level facilities to collect rabies vaccines from a central location. In Sri Lanka, rabies vaccine is delivered from the central level to the lower level facilities. In Cambodia, all three non-private rabies PEP providers work directly with the supplier to have rabies vaccines delivered. Bhutan provides an example of rabies vaccines being successfully incorporated into existing EPI distribution structures while awaiting development of a rabies vaccine-specific distribution network. Even with a separate distribution network, rabies PEP is stored in refrigerators with EPI vaccines at some Bhutan health facilities. Additionally, some Bhutan EPI staff administer rabies vaccine. EPI staff receive extensive training on immunization practices and EPI’s cold storage is closely monitored which provides assurance that the cold chain for rabies PEP remains intact.

There is limited collection and reporting of data on demographics of patients, bite categories, barriers to PEP access and factors affecting adherence to vaccination schedules and completion in most countries. In some cases, this information is collected at the local level. For example, in Cambodia, NIPH clinic collects detailed information on each patient through a comprehensive electronic database [34]. However, this information is not always reported to the central level. While Bhutan provides monitoring tools to health facilities, information is not reported to the central level, and some entities in Cambodia only report on an ad hoc basis, as reporting is not mandatory. This information is needed to understand vaccine uptake and demand, where to allocate the vaccine, and how to improve access to rabies PEP.

Rabies PEP systems in these four countries have several good practices that may be valuable to share. Exploring these systems provides an opportunity to learn and tailor country programs with the aim of increasing access to life-saving rabies prophylaxis. Additionally, rabies PEP management and delivery systems can be adapted from existing systems, such as those of the EPI program. EPI programs often include extensive trainings on administration and cold chain, and standardized guidelines on vaccine introduction, defaulter tracking, and adverse event reporting. These aspects could be leveraged to strengthen rabies PEP distribution systems. There are currently no global standardized protocols for country rabies PEP systems. While unique aspects of countries mean that it is not feasible to implement the same system everywhere, a standardized set of recommendations would help countries to develop effective systems tailored to the country context. Although this assessment focused solely on human rabies PEP, elimination of human rabies deaths cannot be done with rabies PEP alone. Achievement of elimination targets will require the collaborative efforts of the human, animal and other sectors [2,4,9,10,12,35–39].

## Figures and Tables

**Table 1:** Rabies vaccine administration, procurement, and delivery in Bangladesh, Bhutan, Cambodia, and Sri Lanka.

Country	Vaccine brands available^1^ (manufacturer)	Administration route^3^ (year ID introduced)	Schedule^4^	Vaccine in private sector	Procurement frequency	Cold chain^5^	Distribution system^5^	Mandatory reporting^7^	National program
Bangladesh	Rabix-vc (Incepta)	ID (2011)	Updated Thai Red Cross	Available	Annually	Independent	Independent	Yes	Yes
Bhutan	Abhayrab (Human Biologicals Institute)	ID (2012)	Updated Thai Red Cross	Not available	Annually	Independent; EPI^6^ (health facility level only)	Independent	None	Yes
Cambodia	Verorab^2^ (Sanofi Pasteur); Speeda (Liaoning Chengda)	ID and IM (1995)	Updated Thai Red Cross and Essen 5-dose	Widely available	As needed	Independent	Independent	None	None
Sri Lanka	Verorab^2^ (Sanofi Pasteur); Rabipur^2^ (GSK)	ID (1995)	Updated Thai Red Cross	Limited availability	Annually	Independent	Independent	Yes	Yes

1 Vaccine brands available in the public sector in Bangladesh, Bhutan, and Sri Lanka and at Angkor Hospital for Children (AHC), Institut Pasteur du Cambodge (IPC) rabies vaccination center, and National Institutes of Public Health (NIPH) clinic in Cambodia.

2 WHO pre-qualified.

3 ID = Intradermal; IM = Intramuscular.

4 Updated Thai Red Cross (2-site ID route of administration on days 0, 3, 7, 28); Essen 5-dose (1-site IM route of administration on days 0, 3, 7, 14, 28).

5 Independent refers to a system specifically for rabies PEP, not utilizing the EPI system or combining with any other biologicals.

6 EPI = Expanded Programme on Immunization.

7 Reporting on number of vaccine vials used, number of bite patients, number of patients receiving vaccine, or other aspects relating to the use of rabies PEP.
